# Superior Mesenteric Vein Thrombosis Complicating Acute Alcohol-Induced Pancreatitis

**DOI:** 10.7759/cureus.104966

**Published:** 2026-03-10

**Authors:** Kamran Mushtaq, Summiya Nasim, Rizwan Mushtaq, Maryam Sardar, Khalid Ahmad

**Affiliations:** 1 Internal Medicine, Northeast Internal Medicine Associates, LaGrange, USA; 2 Internal Medicine, Parkview Health, Fort Wayne, USA; 3 Internal Medicine, Ayub Medical College, Fort Wayne, USA; 4 Internal Medicine, Azad Jammu Kashmir Medical College, Muzaffarabad, PAK; 5 Medicine, Khyber Medical University, Peshawar, PAK

**Keywords:** acute pancreatitis, alcohol-induced pancreatitis, anticoagulation, splanchnic vein thrombosis, superior mesenteric vein thrombosis

## Abstract

Splanchnic vein thrombosis (SVT) is an uncommon but clinically significant complication of acute pancreatitis, attributed to local inflammation and a transient hypercoagulable state. Superior mesenteric vein (SMV) thrombosis is particularly concerning because of its association with bowel ischemia and long-term portal venous complications. Optimal management strategies remain incompletely defined, particularly regarding the role and timing of anticoagulation. We report the case of a 36-year-old woman with acute alcohol-induced pancreatitis complicated by non-occlusive SMV thrombosis extending toward the portal vein. The diagnosis was established using contrast-enhanced computed tomography (CT) and confirmed by magnetic resonance imaging. The patient was successfully treated with unfractionated heparin followed by transition to a direct oral anticoagulant, with clinical and biochemical improvement. This case highlights the importance of early recognition of vascular complications in acute pancreatitis and supports consideration of anticoagulation in carefully selected patients to prevent thrombus progression and potential ischemic complications.

## Introduction

Acute pancreatitis is a common gastrointestinal disorder with an annual incidence of approximately 13 to 45 cases per 100,000 individuals and a broad spectrum of clinical severity ranging from mild inflammation to severe necrotizing disease [1]. Local vascular complications are increasingly recognized, particularly in moderate to severe cases [2]. Splanchnic vein thrombosis (SVT) may involve the splenic, portal, or superior mesenteric veins, either in isolation or in combination [3]. Although SVT is the most frequently reported form, superior mesenteric vein (SMV) thrombosis is relatively uncommon and less frequently described in the literature.

The underlying pathophysiology is multifactorial and reflects the components of Virchow’s triad, including local inflammatory endothelial injury, venous stasis from compression by peripancreatic edema or collections, and a transient hypercoagulable state associated with systemic inflammation [4]. Vascular complications of the splanchnic circulation have been well described in hepatobiliary and pancreatic inflammatory disorders [5].

Although splenic vein thrombosis is more frequently reported, SMV thrombosis carries greater clinical significance due to its association with bowel ischemia and increased morbidity. The optimal management of pancreatitis-associated SVT remains controversial, particularly regarding anticoagulation. We present a case of acute alcohol-induced pancreatitis complicated by SMV thrombosis and review current evidence guiding anticoagulation in this setting.

## Case presentation

A 36-year-old woman with a medical history of alcohol use disorder, chronic pain syndrome, generalized anxiety disorder, and hypothyroidism presented with acute epigastric abdominal pain associated with nausea and vomiting. She reported daily alcohol consumption for several years, averaging multiple drinks per day, and had experienced a prior episode of pancreatitis approximately six months earlier.

On presentation, the patient was hemodynamically stable. Physical examination revealed epigastric tenderness without peritoneal signs. Initial laboratory evaluation demonstrated a mildly elevated lipase level with leukocytosis. Liver enzymes were mildly elevated, and triglyceride levels were elevated but below the threshold typically associated with hypertriglyceridemia-induced pancreatitis. Renal function and platelet count were normal (Table 1). The diagnosis of acute pancreatitis was established based on characteristic abdominal pain, elevated lipase levels, and confirmatory imaging findings on contrast-enhanced computed tomography (CT).

**Table 1:** Initial Laboratory Findings on Admission Initial laboratory evaluation demonstrated a mildly elevated lipase level and leukocytosis consistent with acute pancreatitis. Liver enzymes were mildly elevated without hyperbilirubinemia. Triglyceride levels were elevated but below the threshold typically associated with hypertriglyceridemia-induced pancreatitis. Renal function and platelet count were preserved at presentation, supporting safe initiation of anticoagulation following the diagnosis of superior mesenteric vein thrombosis.

Laboratory Test	Value	Reference Range
Lipase	83 U/L	11–82
White blood cell count	11.8×10⁹/L	4.0–11.0
Hemoglobin	14.6 g/dL	12.0–16.0
Platelets	253×10⁹/L	150–400
Aspartate aminotransferase	68 U/L	<40
Alanine aminotransferase	59 U/L	<41
Alkaline phosphatase	83 U/L	44–147
Total bilirubin	0.7 mg/dL	0.2–1.2
Triglycerides	267 mg/dL	<150
Creatinine	0.72 mg/dL	0.6–1.2

**Figure 1:**
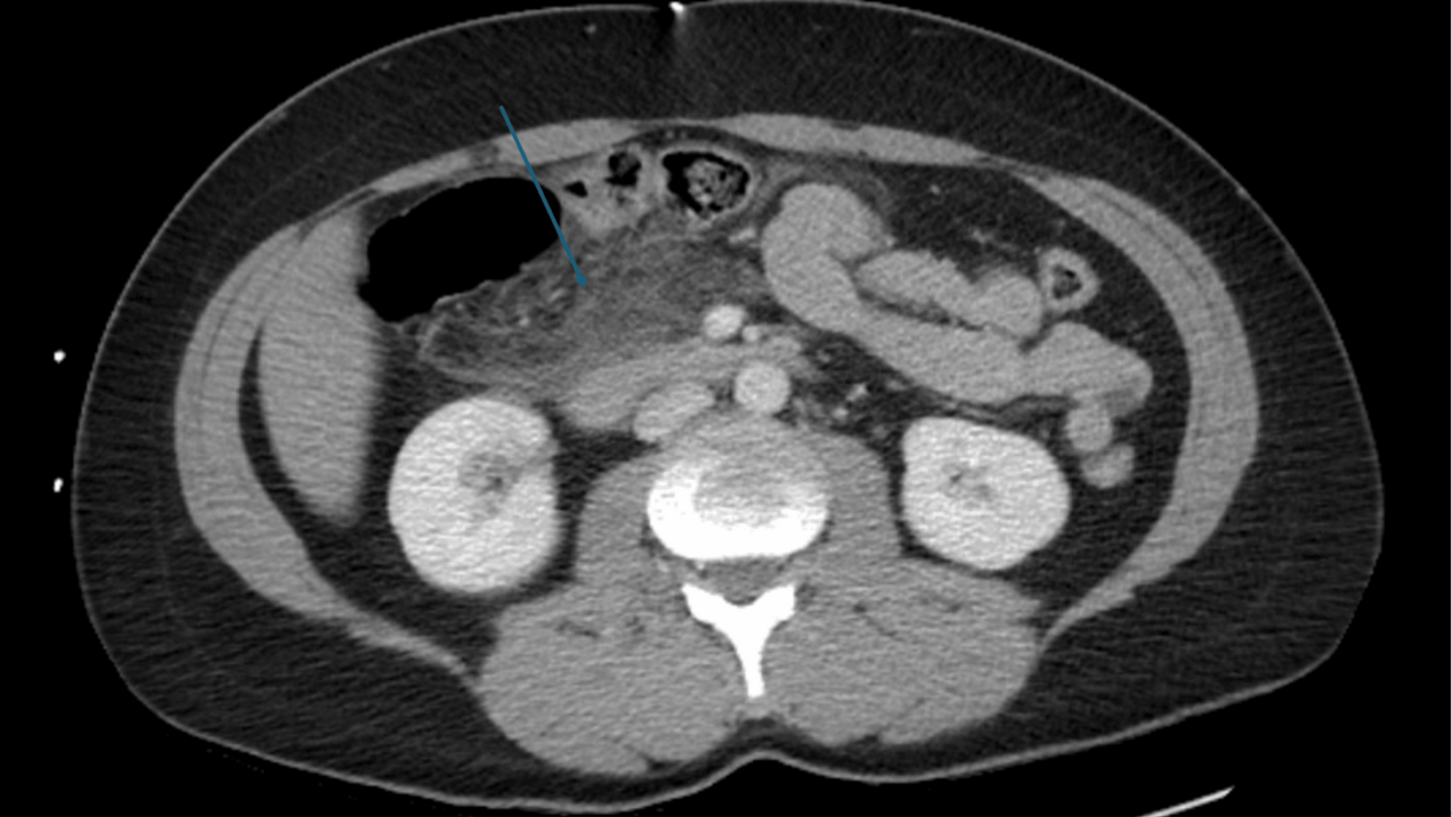
Contrast-Enhanced CT of the Abdomen Demonstrating Acute Pancreatitis Contrast-enhanced CT abdomen demonstrating severe inflammatory changes centered in the pancreatic head and proximal body with associated peripancreatic edema and fluid, consistent with acute interstitial pancreatitis. No pancreatic necrosis or abscess was identified.

Contrast-enhanced CT of the abdomen and pelvis obtained in the portal venous phase demonstrated acute pancreatitis without evidence of necrosis or abscess formation (Figure 1). The patient was managed conservatively with intravenous fluids, bowel rest, analgesia, antiemetics, and proton pump inhibitor therapy.

**Figure 2:**
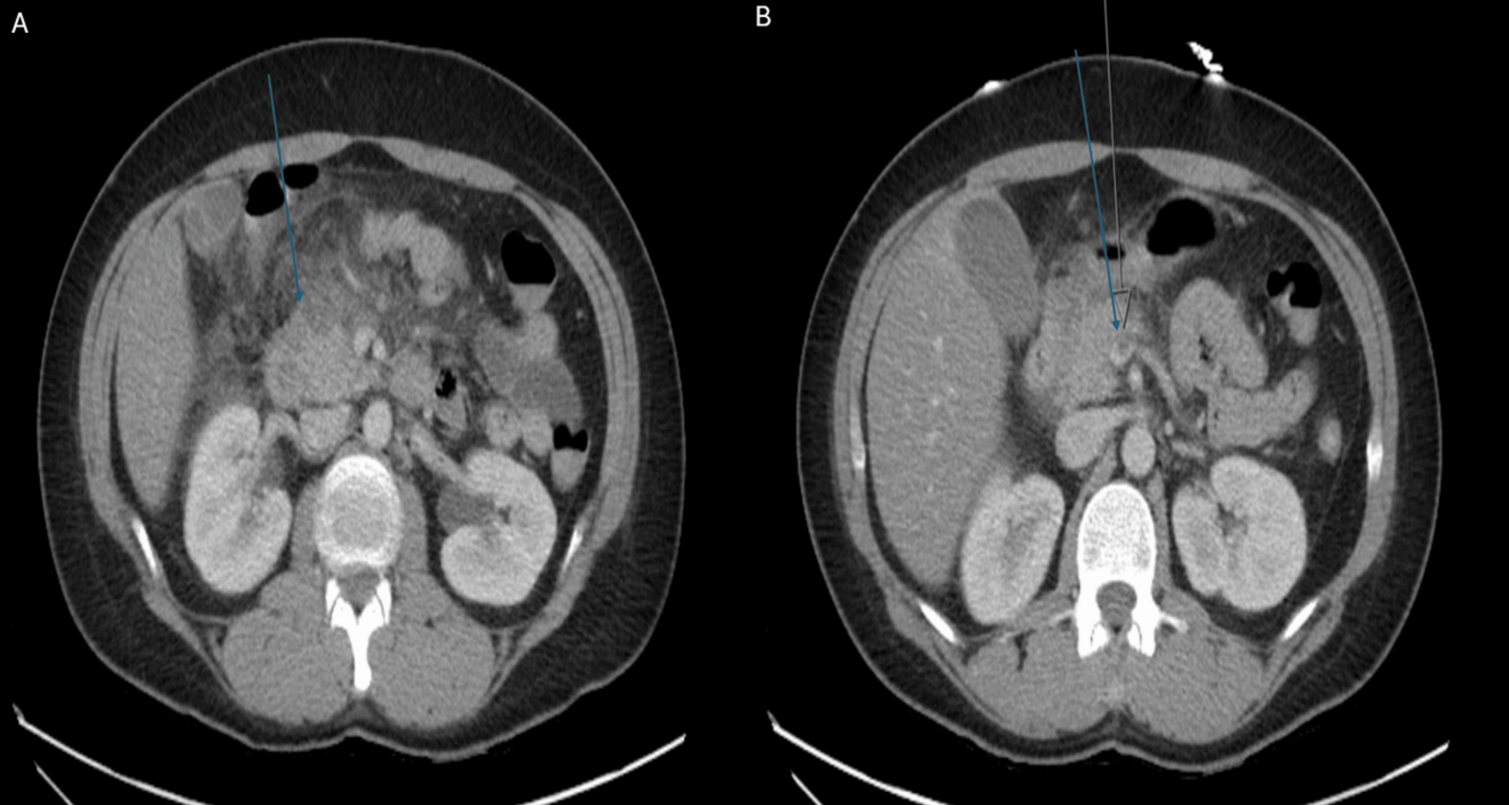
Contrast-Enhanced CT of the Abdomen Demonstrating Acute Pancreatitis and Associated Vascular Complication Contrast-enhanced CT of the abdomen demonstrating inflammatory and vascular complications of acute pancreatitis. (A) CT image showing marked inflammatory enlargement of the pancreatic head with surrounding peripancreatic fat stranding and edema, consistent with acute interstitial pancreatitis. (B) CT image from the same study demonstrating a hypodense filling defect within the superior mesenteric vein, consistent with acute thrombosis.

During hospitalization, she developed fever and persistent abdominal discomfort, prompting repeat imaging. A subsequent contrast-enhanced CT scan demonstrated progressive inflammatory changes of the pancreas and a new hypodense filling defect within the SMV, consistent with acute thrombosis (Figures 2A, 2B).

Given the involvement of the SMV and the potential risk of bowel ischemia, surgical consultation was considered, and the patient was monitored closely for signs of intestinal compromise. The patient was initiated on continuous intravenous unfractionated heparin, titrated to achieve a therapeutic activated partial thromboplastin time. Platelet counts and coagulation parameters remained stable. Over the following 48 hours, her abdominal pain improved, she became afebrile, and lipase levels normalized. She was transitioned to oral apixaban prior to discharge, with a planned anticoagulation duration of three to six months. An outpatient hematology referral was arranged. Inpatient thrombophilia testing was deferred due to the presence of a clear provoking factor.

## Discussion

SVT is a recognized vascular complication of acute pancreatitis, with reported incidence ranging from 1% to 15% depending on disease severity and imaging utilization [1,2]. The development of thrombosis is multifactorial and involves local inflammatory extension, endothelial injury, venous stasis, and compression by peripancreatic edema or collections [3,4]. Vascular involvement of the portal and mesenteric systems has been well described in inflammatory hepatobiliary disorders, further supporting the mechanistic link between acute pancreatic inflammation and venous thrombosis [5].

SMV thrombosis is particularly concerning due to its potential to impair mesenteric venous outflow and lead to bowel ischemia. Although many cases remain clinically stable, progressive thrombosis may result in intestinal edema, reduced perfusion, and, in severe cases, mesenteric ischemia requiring surgical intervention. Early recognition and timely anticoagulation are therefore important to prevent thrombus propagation and potential ischemic complications.

Direct oral anticoagulants have increasingly been evaluated in the treatment of SVT, with long-term data suggesting favorable efficacy and safety profiles in selected noncirrhotic populations [6]. Single-center experiences in pancreatitis-associated SVT demonstrate variable clinical outcomes, with some patients achieving spontaneous recanalization and others developing persistent thrombosis, underscoring the heterogeneity of disease progression [7]. Case-based literature further illustrates that splenic vein thrombosis may occur even in less severe presentations, highlighting the importance of individualized assessment [8].

More focused analyses evaluating anticoagulation specifically in acute pancreatitis-associated SVT have yielded mixed findings. Junare et al. reported no statistically significant difference in major clinical outcomes between anticoagulated and non-anticoagulated groups [9], reflecting ongoing uncertainty in management strategies. However, contemporary reviews emphasize that anticoagulation may be particularly warranted when the portal or SMVs are involved due to the higher risk of bowel ischemia and thrombus propagation [10]. In this context, our case highlights the potential benefit of early anticoagulation in SMV thrombosis to prevent progression and support favorable clinical recovery.

In patients with a transient provoking factor such as acute pancreatitis, anticoagulation for three to six months is generally considered reasonable, with treatment duration tailored to thrombus extent and bleeding risk. Follow-up imaging is commonly recommended to assess thrombus resolution or recanalization, which has been reported in a substantial proportion of patients after anticoagulation therapy. In our patient, early initiation of anticoagulation followed by transition to oral therapy aligns with current literature supporting the prevention of thrombus progression and potential ischemic complications in SMV thrombosis.

## Conclusions

SMV thrombosis is an uncommon but important complication of acute pancreatitis. Early recognition through appropriate imaging and timely initiation of anticoagulation can result in favorable clinical outcomes. Clinicians should also consider repeat imaging when symptoms persist or worsen, as vascular complications may evolve during the disease course. This case reinforces the role of anticoagulation in selected patients with pancreatitis-associated SVT and highlights the need for further prospective studies to guide management.
